# 5-(4-Chloro­phen­oxy)-3-methyl-1-phenyl-1*H*-pyrazole-4-carbaldehyde

**DOI:** 10.1107/S1600536814007879

**Published:** 2014-04-16

**Authors:** N. Vinutha, S. Madan Kumar, S. Shobhitha, B. Kalluraya, N. K. Lokanath, D. Revannasiddaiah

**Affiliations:** a Department of Studies in Physics, University of Mysore, Manasagangotri, Mysore 570 006, India; b Department of Studies in Chemistry, Mangalore University, Mangalagangotri, Mangalore 574 199, India

## Abstract

In the title compound, C_17_H_13_ClN_2_O_2_, the phenyl and chloro­benzene rings are inclined to the central pyrazole ring at 40.84 (9) and 65.30 (9)°, respectively. In the crystal, pairs of C—H⋯π inter­actions link the mol­ecules into inversion dimers and C—H⋯O hydrogen bonds link these dimers into columns extended in [010]. The crystal packing exhibits short inter­molecular O⋯Cl contacts of 3.0913 (16) Å.

## Related literature   

For biological properties and pharmocological applications of ar­yloxy pyrazole derivatives, see: Rai *et al.* (2008 ▶); Girisha *et al.* (2010 ▶); Isloor *et al.* (2009 ▶, 2010 ▶); Shobhitha *et al.* (2013 ▶). For related structures, see: Shahani, Fun, Ragavan *et al.* (2011 ▶); Shahani, Fun, Shetty *et al.* (2011 ▶); Prasath *et al.* (2011 ▶).
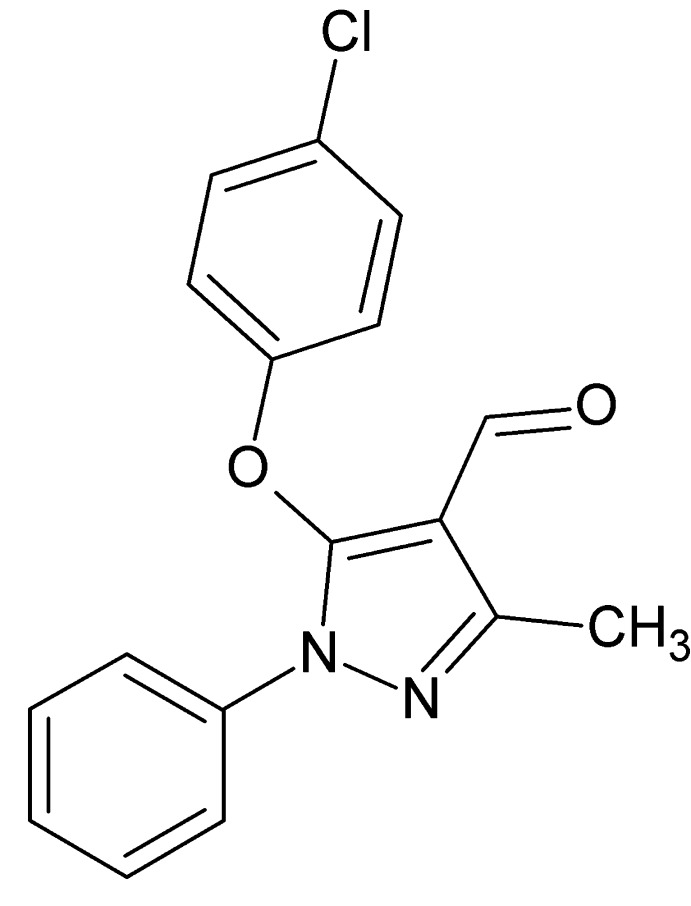


## Experimental   

### 

#### Crystal data  

C_17_H_13_ClN_2_O_2_
*M* *_r_* = 312.74
Monoclinic, 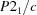

*a* = 9.1016 (7) Å
*b* = 7.5298 (6) Å
*c* = 22.1242 (16) Å
β = 93.908 (3)°
*V* = 1512.7 (2) Å^3^
*Z* = 4
Cu *K*α radiation
μ = 2.31 mm^−1^
*T* = 296 K
0.23 × 0.22 × 0.21 mm

#### Data collection  

Bruker X8 Proteum diffractometer
Absorption correction: multi-scan (*SADABS*; Bruker, 2013 ▶) *T*_min_ = 0.619, *T*_max_ = 0.643
9744 measured reflections
2501 independent reflections
2314 reflections with *I* > 2σ(*I*)
*R*_int_ = 0.041

#### Refinement  

*R*[*F* ^2^ > 2σ(*F* ^2^)] = 0.039
*wR*(*F* ^2^) = 0.105
*S* = 1.03
2501 reflections
200 parameters
H-atom parameters constrained
Δρ_max_ = 0.25 e Å^−3^
Δρ_min_ = −0.38 e Å^−3^

### 

Data collection: *APEX2* (Bruker, 2013 ▶); cell refinement: *SAINT* (Bruker, 2013 ▶); data reduction: *SAINT*; program(s) used to solve structure: *SHELXS97* (Sheldrick, 2008 ▶); program(s) used to refine structure: *SHELXL97* (Sheldrick, 2008 ▶); molecular graphics: *Mercury* (Macrae *et al.*, 2008 ▶); software used to prepare material for publication: *PLATON* (Spek, 2009 ▶).

## Supplementary Material

Crystal structure: contains datablock(s) global, I. DOI: 10.1107/S1600536814007879/cv5447sup1.cif

Structure factors: contains datablock(s) I. DOI: 10.1107/S1600536814007879/cv5447Isup2.hkl

Supporting information file. DOI: 10.1107/S1600536814007879/cv5447Isup3.cml

CCDC reference: 996262

Additional supporting information:  crystallographic information; 3D view; checkCIF report

## Figures and Tables

**Table 1:** Hydrogen-bond geometry (Å, °) *Cg* is the centroid of the C11–C16 ring.

*D*—H⋯*A*	*D*—H	H⋯*A*	*D*⋯*A*	*D*—H⋯*A*
C6—H6⋯O2^i^	0.93	2.58	3.503 (2)	171
C2—H2⋯*Cg* ^ii^	0.93	2.63	3.476 (2)	152

Symmetry codes: (i) 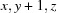; (ii) 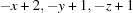.

**Fig. 1.**
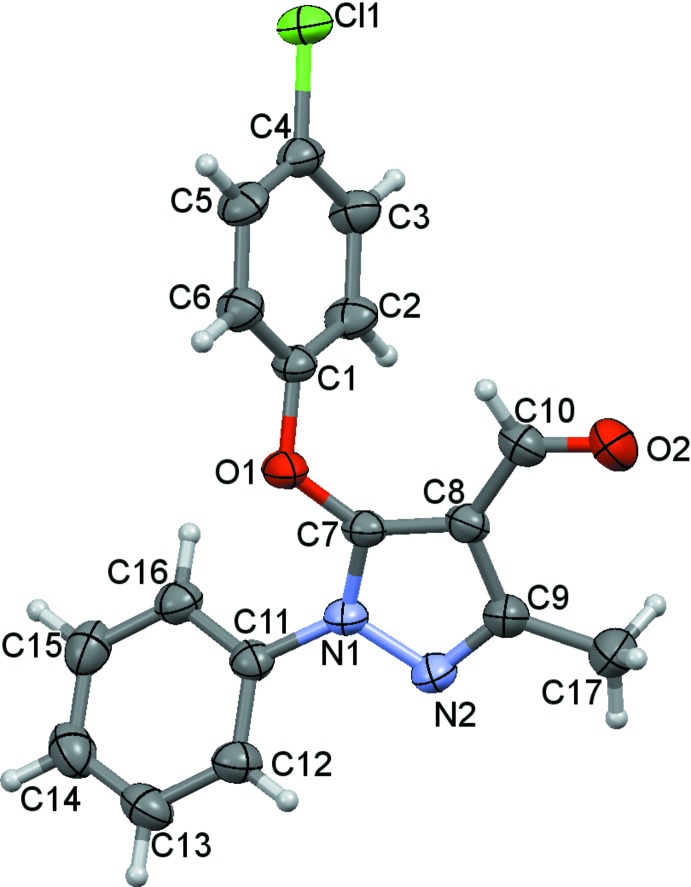
The molecular structure of the title compound showing the atomic numbering and 50% probability displacement ellipsoids.

## supplementary crystallographic information

### 1. Comment

Aryloxy pyrazoles and their derivatives possess a significant pharmcological activities such as antimicrobial (Rai *et al.* 2008; Girisha *et al.*, 2010), anti-inflammatory (Isloor *et al.*, 2009) and analgesic activities (Shobhitha *et al.*, 2013). The title compound can serve as an intermediate in the synthesis of various pyrazole derivatives with significant pharmacological activities (Isloor *et al.*, 2010).

In the title compound (Fig.1), all bond lengths and angles are normal and correspond well to those observed in the related compounds (Shahani, Fun, Ragavan *et al.*, 2011; Shahani, Fun, Shetty *et al.*, 2011; Prasath *et al.*, 2011). The pyrazole ring makes dihedral angles of 65.30 (9)° with chlorobenzene ring and 40.84 (9)° with benzene ring. The dihedral angle between the chlorobenzene ring and benzene ring is 76.23 (9)°.

In the crystal, C–H···π interactions (Table 1) link the molecules into inversion dimers, and intermolecular C–H···O hydrogen bonds (Table 1) link these dimers into columns extended in [010]. The crystal packing exhibits short intermolecular O···Cl contacts of 3.0913 (16) Å.

### 2. Experimental

The title compound was prepared by refluxing a mixture of 5-chloro-3-methyl-1-phenyl-1*H*-pyrazol-4-carboxaldehyde (0.1 mol) and 4-chloro phenol (0.1 mol) in 10 ml of dimethyl sulfoxide. To this solution, 0.1 mol of potassium hydroxide was added. The reaction mixture was refluxed for 3 hrs and then it was cooled to room temperature and poured to crushed ice. The solid product that separated was filtered and dried. It was then recrystallized from ethanol. Crystals suitable for X-ray analysis were obtained from slow evaporation of ethanol.

### 3. Refinement

All the H atoms were fixed geometrically (C—H= 0.93–0.96 Å) and allowed to ride on their parent atoms with *U*_iso_(H) = 1.5*U*_eq_(*C*-methyl) and = 1.2*U*_eq_(C) for other H atoms.

### Crystal data

**Table:**

C_17_H_13_ClN_2_O_2_	*F*(000) = 648
*M**_r_* = 312.74	*D*_x_ = 1.373 Mg m^−^^3^
Monoclinic, *P*2_1_/*c*	Cu *K*α radiation, λ = 1.54178 Å
Hall symbol: -P 2ybc	Cell parameters from 2501 reflections
*a* = 9.1016 (7) Å	θ = 4.0–64.4°
*b* = 7.5298 (6) Å	µ = 2.31 mm^−^^1^
*c* = 22.1242 (16) Å	*T* = 296 K
β = 93.908 (3)°	Block, brown
*V* = 1512.7 (2) Å^3^	0.23 × 0.22 × 0.21 mm
*Z* = 4	

### Data collection

**Table:**

Bruker X8 Proteum diffractometer	2501 independent reflections
Radiation source: Bruker MicroStar microfocus rotating anode	2314 reflections with *I* > 2σ(*I*)
Helios multilayer optics monochromator	*R*_int_ = 0.041
Detector resolution: 18.4 pixels mm^-1^	θ_max_ = 64.5°, θ_min_ = 4.0°
φ and ω scans	*h* = −10→10
Absorption correction: multi-scan (*SADABS*; Bruker, 2013)	*k* = −3→8
*T*_min_ = 0.619, *T*_max_ = 0.643	*l* = −25→25
9744 measured reflections	

### Refinement

**Table:**

Refinement on *F*^2^	Secondary atom site location: difference Fourier map
Least-squares matrix: full	Hydrogen site location: inferred from neighbouring sites
*R*[*F*^2^ > 2σ(*F*^2^)] = 0.039	H-atom parameters constrained
*wR*(*F*^2^) = 0.105	*w* = 1/[σ^2^(*F*_o_^2^) + (0.0555*P*)^2^ + 0.5076*P*] where *P* = (*F*_o_^2^ + 2*F*_c_^2^)/3
*S* = 1.03	(Δ/σ)_max_ < 0.001
2501 reflections	Δρ_max_ = 0.25 e Å^−^^3^
200 parameters	Δρ_min_ = −0.38 e Å^−^^3^
0 restraints	Extinction correction: *SHELXL97* (Sheldrick, 2008), FC^*^=KFC[1+0.001XFC^2^Λ^3^/SIN(2Θ)]^-1/4^
Primary atom site location: structure-invariant direct methods	Extinction coefficient: 0.0171 (10)

### Special details

**Table:**

Geometry. Bond distances, angles *etc*. have been calculated using the rounded fractional coordinates. All su's are estimated from the variances of the (full) variance-covariance matrix. The cell e.s.d.'s are taken into account in the estimation of distances, angles and torsion angles
Refinement. Refinement on *F*^2^ for ALL reflections except those flagged by the user for potential systematic errors. Weighted *R*-factors *wR* and all goodnesses of fit *S* are based on *F*^2^, conventional *R*-factors *R* are based on *F*, with *F* set to zero for negative *F*^2^. The observed criterion of *F*^2^ > σ(*F*^2^) is used only for calculating -*R*-factor-obs *etc*. and is not relevant to the choice of reflections for refinement. *R*-factors based on *F*^2^ are statistically about twice as large as those based on *F*, and *R*-factors based on ALL data will be even larger.

### Fractional atomic coordinates and isotropic or equivalent isotropic displacement parameters (Å^2^)

**Table:**

	*x*	*y*	*z*	*U*_iso_*/*U*_eq_	
Cl1	0.33674 (5)	0.67384 (7)	0.29951 (2)	0.0525 (2)	
O1	0.96938 (13)	0.67414 (15)	0.37256 (6)	0.0417 (4)	
O2	0.96678 (16)	0.16557 (19)	0.27222 (7)	0.0579 (5)	
N1	1.15356 (14)	0.51714 (19)	0.42604 (6)	0.0354 (4)	
N2	1.23196 (16)	0.3599 (2)	0.42482 (7)	0.0420 (5)	
C1	0.81860 (18)	0.6650 (2)	0.35492 (7)	0.0335 (5)	
C2	0.72891 (19)	0.5340 (3)	0.37544 (8)	0.0419 (5)	
C3	0.58011 (19)	0.5360 (3)	0.35785 (8)	0.0417 (5)	
C4	0.52407 (19)	0.6712 (2)	0.32108 (7)	0.0378 (5)	
C5	0.6139 (2)	0.8024 (2)	0.30129 (8)	0.0438 (6)	
C6	0.7637 (2)	0.8000 (2)	0.31799 (8)	0.0403 (6)	
C7	1.04684 (16)	0.5223 (2)	0.38079 (7)	0.0332 (5)	
C8	1.05021 (18)	0.3640 (2)	0.34914 (7)	0.0354 (5)	
C9	1.16849 (19)	0.2680 (2)	0.37941 (8)	0.0398 (5)	
C10	0.9588 (2)	0.3101 (3)	0.29611 (8)	0.0407 (6)	
C11	1.20153 (18)	0.6520 (2)	0.46814 (7)	0.0336 (5)	
C12	1.3510 (2)	0.6767 (2)	0.47974 (8)	0.0407 (6)	
C13	1.4008 (2)	0.8051 (3)	0.52092 (9)	0.0488 (6)	
C14	1.3028 (2)	0.9086 (3)	0.54945 (9)	0.0533 (7)	
C15	1.1529 (2)	0.8822 (3)	0.53819 (8)	0.0495 (6)	
C16	1.10095 (19)	0.7530 (2)	0.49751 (7)	0.0404 (5)	
C17	1.2243 (2)	0.0881 (3)	0.36473 (11)	0.0592 (7)	
H02A	1.30480	0.05740	0.39310	0.0890*	
H2	0.76800	0.44510	0.40090	0.0500*	
H02B	1.25740	0.08840	0.32440	0.0890*	
H3	0.51850	0.44700	0.37070	0.0500*	
H02C	1.14670	0.00270	0.36720	0.0890*	
H5	0.57430	0.89290	0.27670	0.0530*	
H6	0.82560	0.88770	0.30450	0.0480*	
H10	0.89020	0.39080	0.27940	0.0490*	
H12	1.41770	0.60760	0.46010	0.0490*	
H13	1.50150	0.82140	0.52930	0.0590*	
H14	1.33690	0.99660	0.57640	0.0640*	
H15	1.08660	0.95150	0.55800	0.0590*	
H16	1.00030	0.73440	0.49010	0.0480*	

### Atomic displacement parameters (Å^2^)

**Table:**

	*U*^11^	*U*^22^	*U*^33^	*U*^12^	*U*^13^	*U*^23^
Cl1	0.0314 (3)	0.0597 (4)	0.0649 (3)	0.0089 (2)	−0.0083 (2)	−0.0045 (2)
O1	0.0335 (6)	0.0355 (7)	0.0536 (7)	0.0015 (5)	−0.0141 (5)	0.0023 (5)
O2	0.0543 (9)	0.0558 (9)	0.0619 (9)	−0.0033 (6)	−0.0090 (7)	−0.0195 (7)
N1	0.0304 (7)	0.0367 (8)	0.0378 (7)	0.0046 (6)	−0.0069 (5)	−0.0024 (6)
N2	0.0365 (8)	0.0382 (8)	0.0497 (8)	0.0084 (6)	−0.0094 (6)	−0.0035 (6)
C1	0.0294 (8)	0.0379 (9)	0.0321 (8)	0.0039 (6)	−0.0057 (6)	−0.0005 (6)
C2	0.0382 (9)	0.0447 (10)	0.0418 (9)	0.0044 (8)	−0.0038 (7)	0.0129 (7)
C3	0.0342 (9)	0.0454 (10)	0.0456 (9)	0.0018 (7)	0.0038 (7)	0.0068 (8)
C4	0.0316 (9)	0.0442 (10)	0.0368 (8)	0.0072 (7)	−0.0034 (7)	−0.0030 (7)
C5	0.0423 (10)	0.0432 (10)	0.0445 (9)	0.0091 (8)	−0.0071 (8)	0.0099 (8)
C6	0.0390 (10)	0.0379 (10)	0.0435 (9)	0.0020 (7)	−0.0014 (7)	0.0076 (7)
C7	0.0258 (7)	0.0365 (9)	0.0364 (8)	0.0000 (6)	−0.0044 (6)	0.0037 (7)
C8	0.0302 (8)	0.0366 (9)	0.0386 (8)	−0.0034 (7)	−0.0030 (6)	0.0003 (7)
C9	0.0340 (9)	0.0393 (10)	0.0453 (9)	0.0013 (7)	−0.0021 (7)	−0.0040 (7)
C10	0.0360 (9)	0.0442 (11)	0.0409 (9)	−0.0062 (7)	−0.0037 (7)	−0.0020 (8)
C11	0.0350 (9)	0.0340 (9)	0.0308 (8)	0.0012 (6)	−0.0042 (6)	0.0031 (6)
C12	0.0340 (9)	0.0490 (11)	0.0386 (9)	0.0006 (7)	−0.0020 (7)	−0.0009 (7)
C13	0.0440 (10)	0.0539 (12)	0.0468 (10)	−0.0090 (9)	−0.0097 (8)	−0.0019 (8)
C14	0.0658 (13)	0.0454 (12)	0.0467 (10)	−0.0040 (9)	−0.0104 (9)	−0.0069 (8)
C15	0.0623 (12)	0.0430 (11)	0.0429 (9)	0.0136 (9)	0.0021 (8)	−0.0035 (8)
C16	0.0360 (9)	0.0432 (10)	0.0412 (9)	0.0062 (7)	−0.0023 (7)	0.0021 (8)
C17	0.0530 (12)	0.0482 (12)	0.0741 (13)	0.0116 (10)	−0.0122 (10)	−0.0135 (10)

### Geometric parameters (Å, º)

**Table:**

Cl1—C4	1.7390 (18)	C11—C16	1.386 (2)
O1—C1	1.403 (2)	C12—C13	1.384 (3)
O1—C7	1.3490 (19)	C13—C14	1.370 (3)
O2—C10	1.214 (3)	C14—C15	1.384 (3)
N1—N2	1.384 (2)	C15—C16	1.386 (3)
N1—C7	1.347 (2)	C2—H2	0.9300
N1—C11	1.426 (2)	C3—H3	0.9300
N2—C9	1.320 (2)	C5—H5	0.9300
C1—C2	1.377 (3)	C6—H6	0.9300
C1—C6	1.377 (2)	C10—H10	0.9300
C2—C3	1.384 (2)	C12—H12	0.9300
C3—C4	1.379 (3)	C13—H13	0.9300
C4—C5	1.373 (2)	C14—H14	0.9300
C5—C6	1.388 (3)	C15—H15	0.9300
C7—C8	1.384 (2)	C16—H16	0.9300
C8—C9	1.425 (2)	C17—H02A	0.9600
C8—C10	1.449 (2)	C17—H02B	0.9600
C9—C17	1.490 (3)	C17—H02C	0.9600
C11—C12	1.380 (2)		
			
C1—O1—C7	119.23 (12)	C13—C14—C15	119.99 (19)
N2—N1—C7	110.91 (13)	C14—C15—C16	120.39 (18)
N2—N1—C11	119.12 (13)	C11—C16—C15	118.86 (16)
C7—N1—C11	129.64 (14)	C1—C2—H2	120.00
N1—N2—C9	105.30 (13)	C3—C2—H2	120.00
O1—C1—C2	122.29 (14)	C2—C3—H3	120.00
O1—C1—C6	115.95 (14)	C4—C3—H3	120.00
C2—C1—C6	121.69 (16)	C4—C5—H5	120.00
C1—C2—C3	119.37 (18)	C6—C5—H5	120.00
C2—C3—C4	119.31 (18)	C1—C6—H6	121.00
Cl1—C4—C3	119.12 (13)	C5—C6—H6	121.00
Cl1—C4—C5	119.89 (13)	O2—C10—H10	118.00
C3—C4—C5	121.00 (16)	C8—C10—H10	118.00
C4—C5—C6	120.08 (15)	C11—C12—H12	120.00
C1—C6—C5	118.54 (15)	C13—C12—H12	120.00
O1—C7—N1	117.94 (14)	C12—C13—H13	120.00
O1—C7—C8	133.72 (14)	C14—C13—H13	120.00
N1—C7—C8	108.15 (13)	C13—C14—H14	120.00
C7—C8—C9	103.96 (14)	C15—C14—H14	120.00
C7—C8—C10	128.28 (16)	C14—C15—H15	120.00
C9—C8—C10	127.72 (15)	C16—C15—H15	120.00
N2—C9—C8	111.66 (14)	C11—C16—H16	121.00
N2—C9—C17	120.29 (16)	C15—C16—H16	121.00
C8—C9—C17	128.05 (16)	C9—C17—H02A	109.00
O2—C10—C8	123.73 (18)	C9—C17—H02B	109.00
N1—C11—C12	118.14 (14)	C9—C17—H02C	109.00
N1—C11—C16	120.96 (14)	H02A—C17—H02B	110.00
C12—C11—C16	120.89 (15)	H02A—C17—H02C	109.00
C11—C12—C13	119.43 (16)	H02B—C17—H02C	109.00
C12—C13—C14	120.43 (17)		
			
C7—O1—C1—C2	36.1 (2)	C2—C3—C4—C5	0.6 (3)
C7—O1—C1—C6	−146.92 (15)	Cl1—C4—C5—C6	−179.47 (13)
C1—O1—C7—N1	−143.39 (14)	C3—C4—C5—C6	0.3 (3)
C1—O1—C7—C8	42.4 (2)	C4—C5—C6—C1	−0.6 (2)
C7—N1—N2—C9	−1.81 (18)	O1—C7—C8—C9	174.01 (17)
C11—N1—N2—C9	−175.88 (14)	O1—C7—C8—C10	−3.9 (3)
N2—N1—C7—O1	−174.08 (13)	N1—C7—C8—C9	−0.60 (17)
N2—N1—C7—C8	1.51 (18)	N1—C7—C8—C10	−178.50 (16)
C11—N1—C7—O1	−0.8 (2)	C7—C8—C9—N2	−0.55 (19)
C11—N1—C7—C8	174.78 (15)	C7—C8—C9—C17	−179.41 (17)
N2—N1—C11—C12	37.2 (2)	C10—C8—C9—N2	177.37 (17)
N2—N1—C11—C16	−141.54 (15)	C10—C8—C9—C17	−1.5 (3)
C7—N1—C11—C12	−135.63 (17)	C7—C8—C10—O2	−177.79 (18)
C7—N1—C11—C16	45.7 (2)	C9—C8—C10—O2	4.8 (3)
N1—N2—C9—C8	1.42 (19)	N1—C11—C12—C13	−179.23 (16)
N1—N2—C9—C17	−179.62 (16)	C16—C11—C12—C13	−0.5 (2)
O1—C1—C2—C3	177.88 (16)	N1—C11—C16—C15	179.80 (15)
C6—C1—C2—C3	1.0 (3)	C12—C11—C16—C15	1.1 (2)
O1—C1—C6—C5	−177.13 (15)	C11—C12—C13—C14	−0.8 (3)
C2—C1—C6—C5	−0.1 (3)	C12—C13—C14—C15	1.5 (3)
C1—C2—C3—C4	−1.3 (3)	C13—C14—C15—C16	−0.9 (3)
C2—C3—C4—Cl1	−179.59 (14)	C14—C15—C16—C11	−0.4 (3)

### Hydrogen-bond geometry (Å, º)

Cg is the centroid of the C11–C16 ring.

**Table:**

*D*—H···*A*	*D*—H	H···*A*	*D*···*A*	*D*—H···*A*
C6—H6···O2^i^	0.93	2.58	3.503 (2)	171
C2—H2···*Cg*^ii^	0.93	2.63	3.476 (2)	152

Symmetry codes: (i) *x*, *y*+1, *z*; (ii) −*x*+2, −*y*+1, −*z*+1.
